# Comparison of CT-based migration analysis against radiostereometric analysis for assessing two-year migration in an uncemented medial pivot tibial knee implant: clinical implications from a prospective safety study

**DOI:** 10.1302/2633-1462.710.BJO-2026-0123.R1

**Published:** 2026-10-16

**Authors:** Lars Harald William Engseth, Charles Jahren Conrad, Frank-David Øhrn, Anselm Schulz, Stephan Maximillian Herbert Röhrl

**Affiliations:** 1 Division of Orthopaedic Surgery, Oslo University Hospital Ullevål, Oslo, Norway; 2 Faculty of Medicine, University of Oslo, Oslo, Norway; 3 Orthopaedic Department, Møre and Romsdal Hospital, Møre and Romsdal Hospital Trust, Ålesund, Norway; 4 Faculty of Medicine and Health Sciences, Department of Neuromedicine and Movement Science (INB), NTNU Norwegian University of Science and Technology, Trondheim, Norway; 5 Department of Radiology and Nuclear Medicine, Oslo University Hospital Ullevål, Oslo, Norway

**Keywords:** Arthroplasty, CTMA, CT-RSA, Diagnostic imaging, Implant migration, Medial pivot, Precision, Radiation dose, Radiostereometric analysis, RSA, radiostereometric analysis (RSA), Knee implant, tibial implants, model-based radiostereometric analysis (RSA), Forgotten Joint Score (FJS-12), orthopaedic implants, Osteoarthritis, Knee Injury, radiographs, medial pivot knee

## Abstract

**Aims:**

Radiostereometric analysis (RSA) is the gold standard for migration analysis of orthopaedic implants, but CT-based RSA has shown comparable results in an increasing number of joints. In this study, we compared the method in an uncemented medial pivot tibial implant. Our primary outcome measure was maximum total point motion (MTPM). Secondary measures were segmental and feature point migration parameters, range of motion (ROM), Forgotten Joint Score (FJS-12), Knee Injury and Osteoarthritis Score (KOOS), effective radiation doses, and time usage.

**Methods:**

A total of 31 patients were prospectively included to receive an uncemented medial pivot knee implant using mechanical alignment (MA) with conventional instruments. Migrations were measured up to 24 months with marker-based RSA, model-based RSA, and CT-based RSA. Clinical data were measured using a goniometer and patient-reported outcome measures ((PROMs); FJS-12 and KOOS).

**Results:**

Migrations at 24 months for MTPM showed a mean of 1.09 mm (95% CI 0.78 to 1.40) for marker-based RSA, 1.17 mm (95% CI 0.85 to 1.49) for model-based RSA, and 0.74 mm (95% CI 0.45 to 1.04) for CT-based RSA. MTPM for CT-based RSA was lower than both RSA methods (p < 0.001). Differences increased between the methods at all timepoints, but migrations stabilized for all methods. Time usage (min:sec) for performing RSA radiographs was 17:02 and 4:10 for CT. FJS-12 score increased from 12.3 at baseline to 70.4 at 24 months; and KOOS increased for all sub-scales. ROM increased by 12.4° (95% CI 5.6 to 19.3).

**Conclusion:**

For clinical importance, the uncemented GMK Sphere shows a stable migration pattern using all methods and can therefore be considered safe. CT-based RSA shows lower migration values of an uncemented medial pivot tibial implant up to 24 months compared with conventional RSA.

Cite this article: *Bone Jt Open* 2026;7(10):1330–1341.

## Introduction

Total knee arthroplasty (TKA) is the standard treatment for end-stage knee osteoarthritis. Despite this, 20% to 25% of patients report dissatisfaction with the outcome.^1^ Research utilizing radiostereometric analysis (RSA) has demonstrated a correlation between early implant migration and revision surgery due to aseptic loosening,^2,3^ which has made it the gold standard for assessing orthopaedic implant migration. RSA can be performed using two methods: marker-based and model-based RSA. Marker-based RSA, using tantalum markers for all analysis, is the gold standard. However, model-based RSA, which uses computer-aided design (CAD) models, is a widely used alternative.^4^

In recent years, CT-based RSA migration analysis methods have emerged. These methods have shown comparable results with RSA in many joints,^5-7^ including its efficacy through precision and accuracy studies.^8,9^ However, CT-based RSA has shown lower migration compared with RSA methods at 12 months.^10^ Therefore, further comparison of CT-based RSA against conventional RSA in clinical migration studies is necessary.

Native knee kinematics are complex,^11^ where ‘roll back’ of the femoral condyle is greater laterally than medially.^12^ Roll back is important for high degrees of flexion. The most common knee implant, the cruciate-retaining implant, does not reproduce roll back.^13^ The medial pivot (MP) knee implant aspires to reproduce native knee kinematics with a ‘ball-in-socket’ medial articulation to maintain medial stability, while it has a low constrained lateral articulation to allow for lateral roll back.^14^ The GMK Sphere (Medacta International, Switzerland) is a MP knee implant design.

Implant loosening is a leading cause of revision surgery.^15^ Loosening could be due to implant design and kinematic flaws, but also implant-bone fixation problems, making secure fixation paramount. Cemented fixation of TKA implants provides lasting stability for up to 15 to 20 years.^16^ However, questions on whether uncemented TKA could improve long-term outcome in young patients have been raised.^17,18^ At the same time TKA use is expected to increase in the younger, active population.^19^ The theoretical advantages of uncemented TKA are bone stock preservation, cement debris protection, and biological fixation to bone.^18^ Therefore, a thorough clinical evaluation is needed for the new uncemented MP implant.

The primary objective of this study was to evaluate migration, measured by maximum total point motion (MTPM) for RSA in a new uncemented MP tibial implant, to validate safety and in vivo stability of the implant. Secondary objectives included comparing MTPM for CT-based RSA, assessing segmental and feature point migration parameters by all methods, range of motion (ROM), patient-reported outcome measures (PROMs); (Knee Injury and Osteoarthritis Score (KOOS),^20^ Forgotten Joint Score (FJS-12)),^21^ effective radiation doses, and time usage. We hypothesized that the implant would show safe levels of migration and that there would be no significant difference in MTPM between the methods.

## Methods

A single group of patients with symptomatic end-stage knee osteoarthritis were prospectively recruited from the out-patient clinic at Oslo University Hospital (OUH), Ullevål, from September 2021 to October 2022.^10^ Patients received an uncemented GMK Sphere TKA and a cemented GMK patella (Medacta International) through a medial parapatellar approach with measured resection technique, resection of both cruciate ligaments, and mechanical alignment without the use of a tourniquet. Palacos R + G bone cement (Heraeus Medical, Germany) was used for the patella. Four to six Ø1 mm tantalum beads (RSA Biomedical, Sweden) were placed in the insert, and five to eight in the tibial bone. All surgeries were performed by the last author (SMR) who had prior experience with the implant.^22^

Two patients were excluded from the study, one due to perioperative conversion to a condylar constrained implant due to ligament insufficiency, the other due to revision for assumed aseptic loosening at eight months. One patient performed 12 months CT-based RSA but did not meet for RSA examinations. Another patient had the wrong knee examined by CT at 12 months. A total of 29 patients met for 24-month follow-up, but one patient was excluded as RSA was not possible to analyze at 24 months. CONSORT guidelines were followed (Figure 1).^23^

**Fig. 1:**
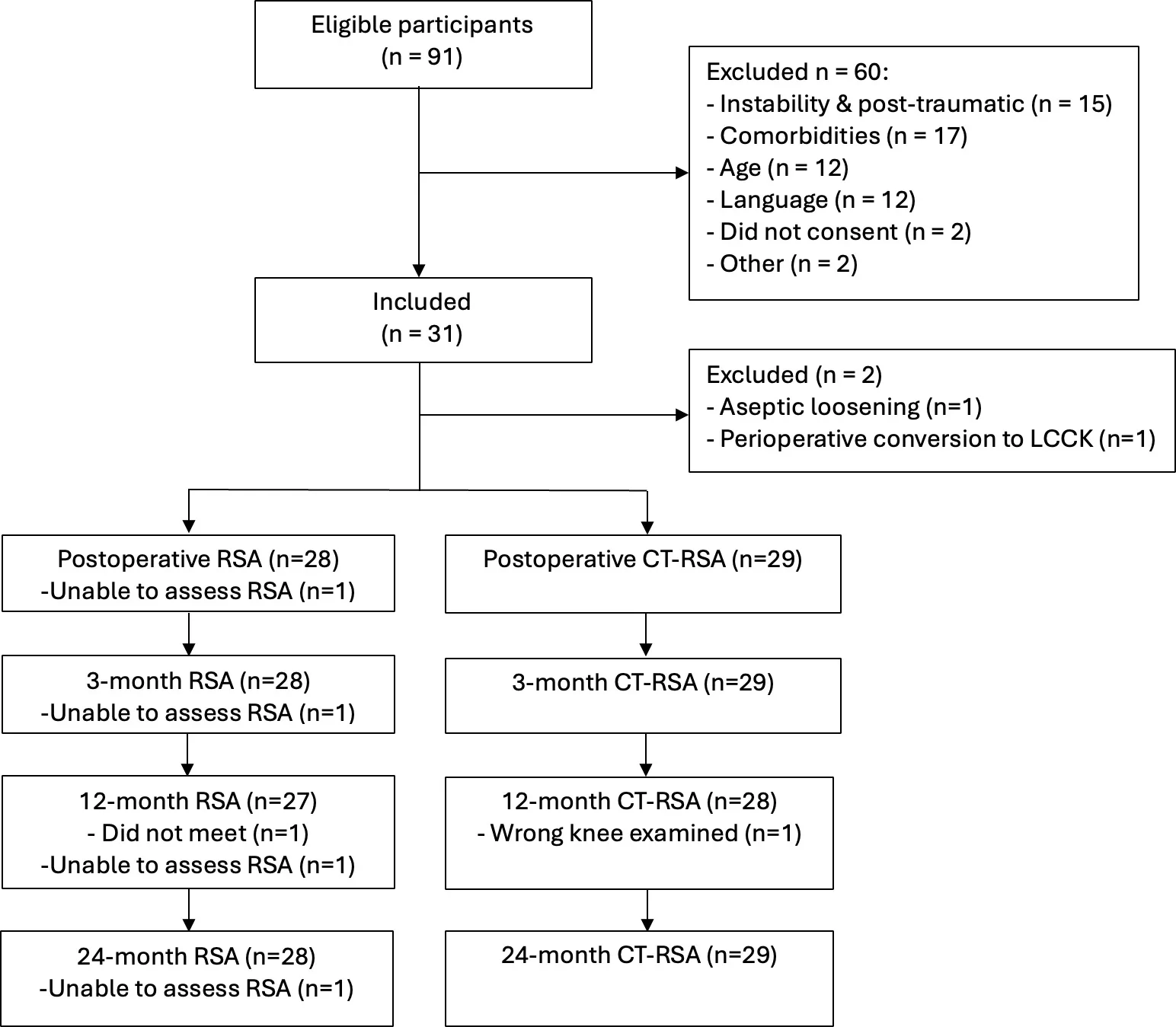
Flowchart. CT-RSA, CT-based radiostereometric analysis; LCCK, NexGen Legacy Constrained Condylar Knee implant (Zimmer Biomet, USA).

### Radiology

Conventional radiographs, RSA, and CT exposures were performed as described in Table I. Tibial implant rotation was evaluated with a modified Berger’s method,^24^ where we found the centre of the tibial bone using rectangle and crossing diagonals (Figure 2). Berger rotation signifies the degrees of internal rotation of the tibial component axis (perpendicular to the posterior aspect of the tibial implant) from the tibial tubercule axis, measured from the centre of the tibial bone.^24,25^ It is also reported as difference to -18° as this is the normal internal rotation.^25^ Berger’s method, hip-knee-ankle angle (HKA), and medial proximal tibia angle (MPTA) were analyzed at three months. CT and RSA time was calculated from the time the patient was called in from the waiting room to when the patient left the examination room (Table II).

**Table I.** Radiology schedule.

Modality	Preoperative	Postoperative	3 months	12 months	24 months
Radiograph	X	X	X	X	X
HKA radiograph	X		X		
CT-based RSA		X	X	X	X
RSA		X	X	X	X

Overview of timepoints for radiological evaluations.

HKA, hip-knee-ankle angle; RSA, radiostereometric analysis.

**Fig. 2:**
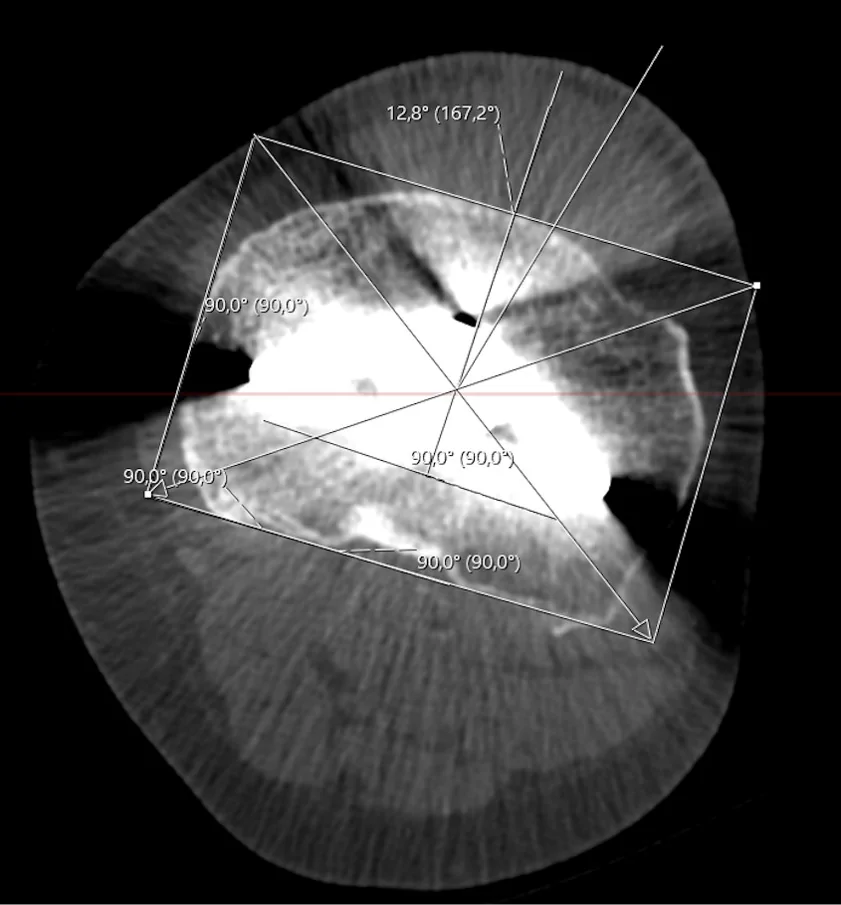
Modified Berger’s method. Sectra IDS7 software (Sectra AB, Sweden) does not have an automatic recognition of the centre of the tibia. The centre was found by creating a rectangle around the tibia and drawing diagonals. The crossing point of the diagonals was defined as the centre.

**Table II.** Demographic and radiological parameters.

Variable	Number
Total patients analyzed	28, 3 excluded
Mean age, yrs (SD)	63.4 (7.2)
Sex, M:F (% female)	11:17 (60.7)
Mean BMI, kg/m^2^ (SD)	29.8 (3.5)
Side, L:R	17:11
Mean time to 3-mth follow-up, mths (SD)	2.8 (0.4)
Mean time to 12-mth follow-up, mths (SD)	12.6 (1.5)
Mean time to 24-mth follow-up, mths (SD)	24.5 (1.7)
Mean HKA (< 180° = varus) (SD)	179.0 (2.3)
Mean MPTA (< 90° = varus) (SD)	90.8 (1.5)
Mean Berger rotation, ° (SD)	15.6 (8.3)
Mean Berger (difference to -18°), ° (SD)	-2.4 (8.3)
Mean CT time, min:sec (SD)	04:10 (1:46)
Mean RSA time, min:sec (SD)	17:02 (6.55)

HKA, postoperative hip-knee-ankle angle; MPTA, postoperative medial proximal tibia angle; RSA, radiostereometric analysis.

### RSA protocol

RSA exposures were performed at zero, three, 12, and 24 months using ceiling-mounted X-ray tubes (Proteus XR/A (GE Healthcare, USA)), Canon Triathlon T3 (Canon Inc, Japan) and knee cage number 10 (UmRSA, RSA Biomedical, Sweden) (Figure 1).^10^ Postoperatively, double examinations were performed for precision measurements for all patients after ambulation and before discharge. Both X-ray tubes had parameters of 133 kV and 6.3 mAs. RSA scenes were analyzed using RSAcore 4.2 (Leiden University Medical Center, the Netherlands) with the ‘consistent-marker’ method.^26^ Migrations were reported for MTPM, segmental and peripheral translation, and rotation for six degrees of freedom. Values for left knees were converted to right values. A maximum condition number (CN) of 120 and mean error (ME) of 0.35 mm, were accepted.^27^ For model-based RSA computer-aided design (CAD), models with predefined peripheral points were used (Figure 3). Radiation exposure was recorded (dose area product, DAP) and effective radiation doses were estimated using a knee conversion factor (0.000009).^28^

**Fig. 3:**
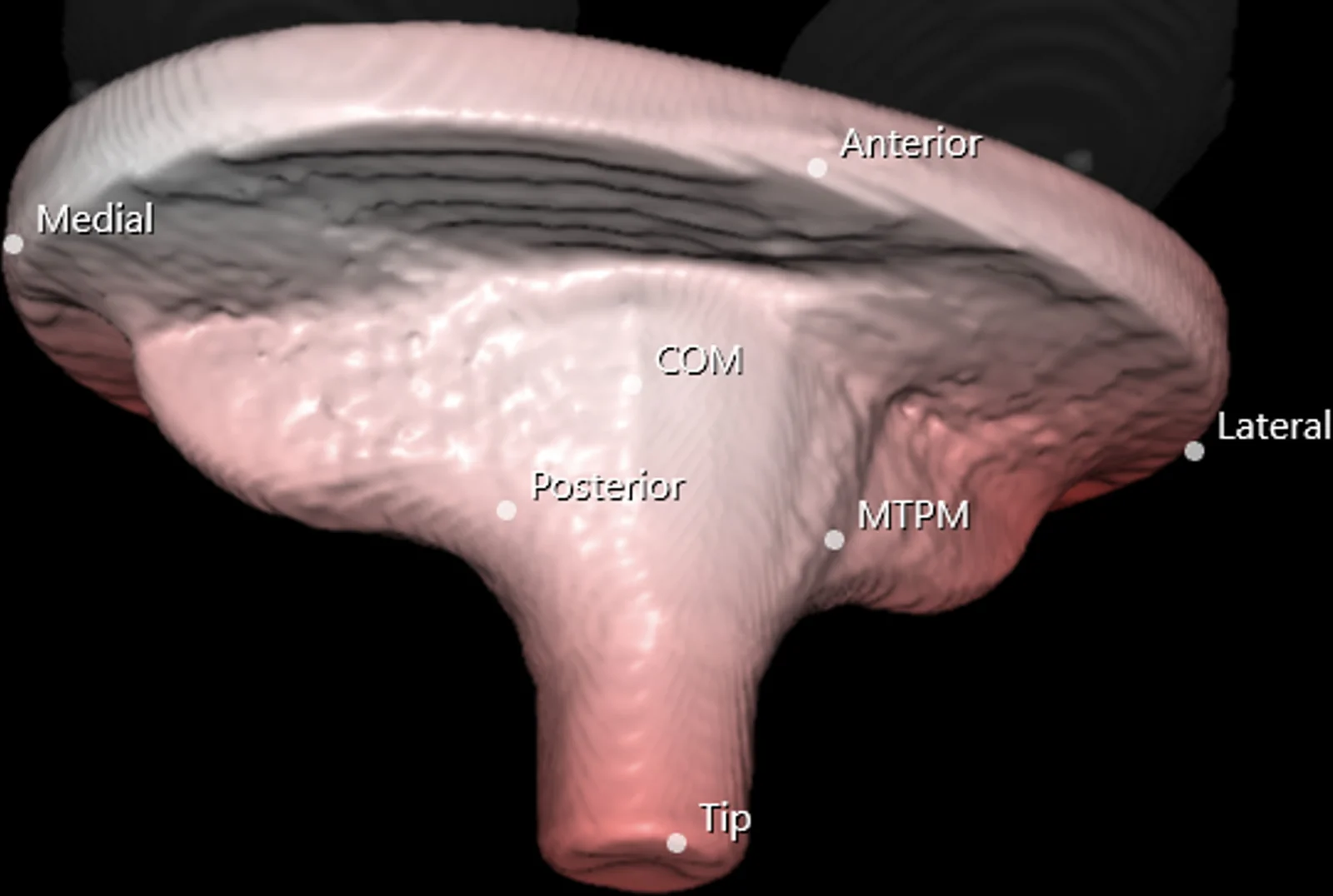
Sectra model (Sectra, Sweden) with peripheral points were created in CT-based radiostereometric analysis (RSA), comparing with peripheral points in the computer-aided design models of the tibial implant from RSAcore. COM, centre of mass; MTPM, maximum total point motion.

### CT-based RSA protocol

CT examinations were performed using a Siemens SOMATOM Drive (Siemens Healthcare, Germany) with metal artefact reduction (MAR) protocol (Table III).^7,10^ Effective doses were estimated by multiplying dose length product (DLP) with the knee conversion factor 0.0004 mSv/(mGy*cm).^29^ Double examinations were performed as for RSA. Scans were examined by segmentation (Figure 4) using the CT-based micromotion analysis software (CTMA, v. 25.2, Sectra, Sweden).^7^

**Table III.** CT protocol parameters.

Variable	Results
Tube voltage, kV	120
Tube current, mAs	100
Slice thickness, mm	0.625
Rotation time, s	0.5
Pitch	1
Field of view, mm	200 × 200
Effective dose, mSv	0.071

**Fig. 4:**
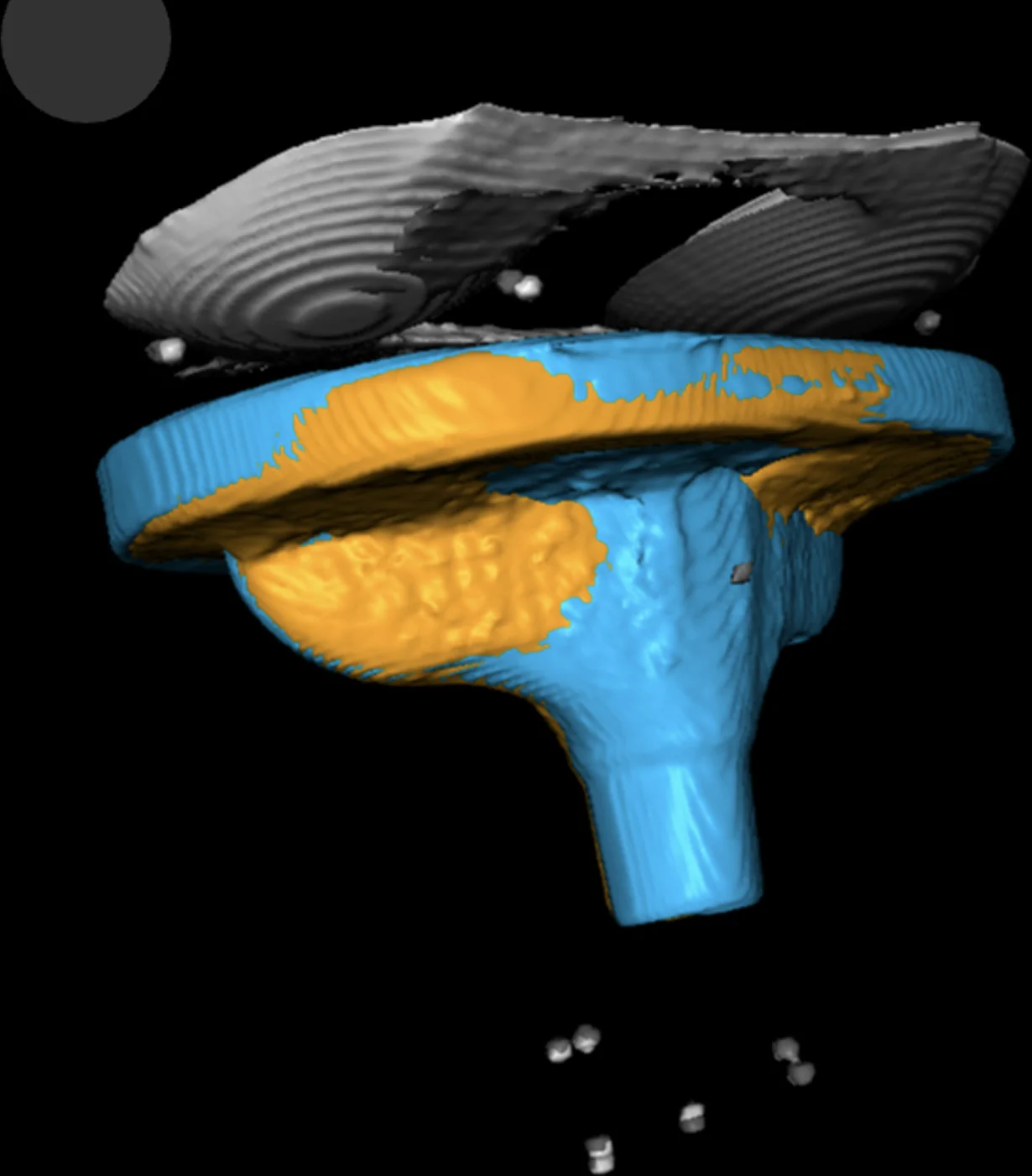
Segmentation in CT-based radiostereometric analysis.

### Differences between RSA and CT-based RSA

CT values were converted to fit with RSA coordinates using STATA v. 18.5 (StataCorp LLC, USA).^30^ RSA and CT coordinate systems were aligned with clinically relevant terms: medial, proximal, anterior translations, and transversal, internal, and varus rotations.^5^ Segmental translations were calculated using the centre of mass (COM) as a reference point (Figure 3). Peripheral points of the RSA CAD model were manually created in CTMA by copying the CAD model (Figure 3).

### Outcome measures

The primary outcome measure was MTPM measured at 24 months by RSA methods (marker-based RSA and model-based RSA); and comparing the migration to clinically relevant migration of the new uncemented MP implant. Secondarily, RSA methods were compared with CT-based RSA where the secondary outcome measures were: 1) the difference in precision between methods; 2) differences in segmental and peripheral point migrations between methods; 3) baseline and two-year ROM and PROMs; 4) effective radiation dosages; and 5) differences in time usage between methods.

### Ethics

The Regional Committees for Medical and Health Research Ethics (REK) approved the study (reference 2014/1075/REK Vest) and the protocol is available at clinicaltrials.org (NCT 04017533).^10^ Written consent from the patients was obtained preoperatively. Grants from Sophies Minde, Norway, and salary from Oslo University Hospital, Ullevål, Norway funded the study. Implants were funded through TTO Inven2. CONSORT guidelines and the Declaration of Helsinki have been followed.^23,31^

### Statistical analysis

STATA v. 18.5 was used for analyses. Differences in migration between methods were analyzed using a linear mixed model (LMM). Differences in clinical data between follow ups were analyzed using paired *t*-tests. Missing data was not imputed. Bland–Altman plots were used to assess agreement between methods. Sample size analyses were performed based on MTPM at 24 months’ follow-up. An increase of MTPM of 0.2 mm from 12 to 24 months is previously defined as clinically relevant.^2^ With a power (1-β) of 80%, a significance threshold (α) of 0.05, and an assumed SD of 0.3 based on other studies, a minimum of 20 patients were needed. Overall, 31 patients were included to account for loss to follow-up.

## Results

### Quality measurements

HKA radiographs showed slight varus alignment postoperatively (179°) and medial proximal tibial angle (MPTA) of 90.8° postoperatively (Table II). A modified Berger’s approach showed good rotational alignment of the tibial implant (Table II). One patient was excluded due to high CN and mean error (ME) at 24 months (Table IV).

**Table IV.** Quality parameters of radiostereometric analysis (means) at 24 months.

Variable	Value (SD)
Marker-based RSA	
CN implant	35.8 (14.0)
ME implant, mm	0.13 (0.06)
**Marker-based RSA and model-based RSA**	
CN tibia	56.1 (20.3)
ME tibia, mm	0.26 (0.06)

CN, condition number; MBRSA, model-based RSA; ME, mean error; RSA, radiostereometric analysis.

### Migration

At 24 months MTPM (mm) showed a mean of 1.09 (95% CI 0.78 to 1.40) for marker-based RSA, 1.17 (95% CI 0.85 to 1.49) for model-based RSA, and 0.74 (95% CI 0.45 to 1.04) for CT-based RSA (Table V). MTPM for CT-based RSA was lower than both RSA methods (p < 0.001, linear mixed model). Migrations measured by all methods were stable between 12 and 24 months. Migration analyses showed that the tibial implant migrated early (first three months) but stabilized from about 12 months (Figure 5). Only one patient showed high migration between all RSA methods (Figure 6). CT-based RSA shows a small medial translation not shown using RSA methods (Table V). Marker-based RSA and CT-based RSA show that the implant externally rotates, whereas model-based RSA does not (Table V). All methods show that the prosthesis subsides (Table V). Peripheral points for model-based RSA and CT-based RSA differed in three out of 15 metrics (Table VI).

**Table V.** Segmental migration values for marker-based radiostereometric analysis (RSA) (n = 28), model-based RSA (n = 28), and CT-based RSA (n = 29) at 24 months.

Variable	Marker-based RSA	Model-based RSA	CT-based RSA
**Mean MTPM, mm (95% CI)**	1.09 (0.78 to 1.40)*	1.17 (0.85 to 1.49)*	0.74 (0.45 to 1.04)
**Mean translation, mm (95% CI)**			
Medial	-0.02 (-0.18 to 0.14)†	0.02 (-0.10 to 0.15)*	0.12 (0.08 to 0.17)
Proximal	-0.26 (-0.42 to –0.09)	-0.23 (-0.37 to –0.09)	-0.16 (-0.28 to –0.04)
Anterior	-0.12 (-0.37 to 0.13)	0.05 (-0.09 to 0.19)	0.00 (-0.05 to 0.06)
**Mean rotation, ° (95% CI)**			
Transversal	-0.49 (-1.04 to 0.05)	-0.27 (-0.79 to 0.24)	-0.21 (-0.66 to 0.23)
Internal	-0.29 (-0.48 to –0.10)	-0.07 (-0.41 to 0.27)†	-0.35 (-0.51 to –0.18)
Varus	0.01 (-0.23 to 0.25)	-0.06 (-0.31 to 0.19)	0.08 (-0.05 to 0.20)

* p < 0.010 compared with CT-based RSA.

† p < 0.050 compared with CT-based RSA.

MTPM, maximum total point motion.

**Fig. 5:**
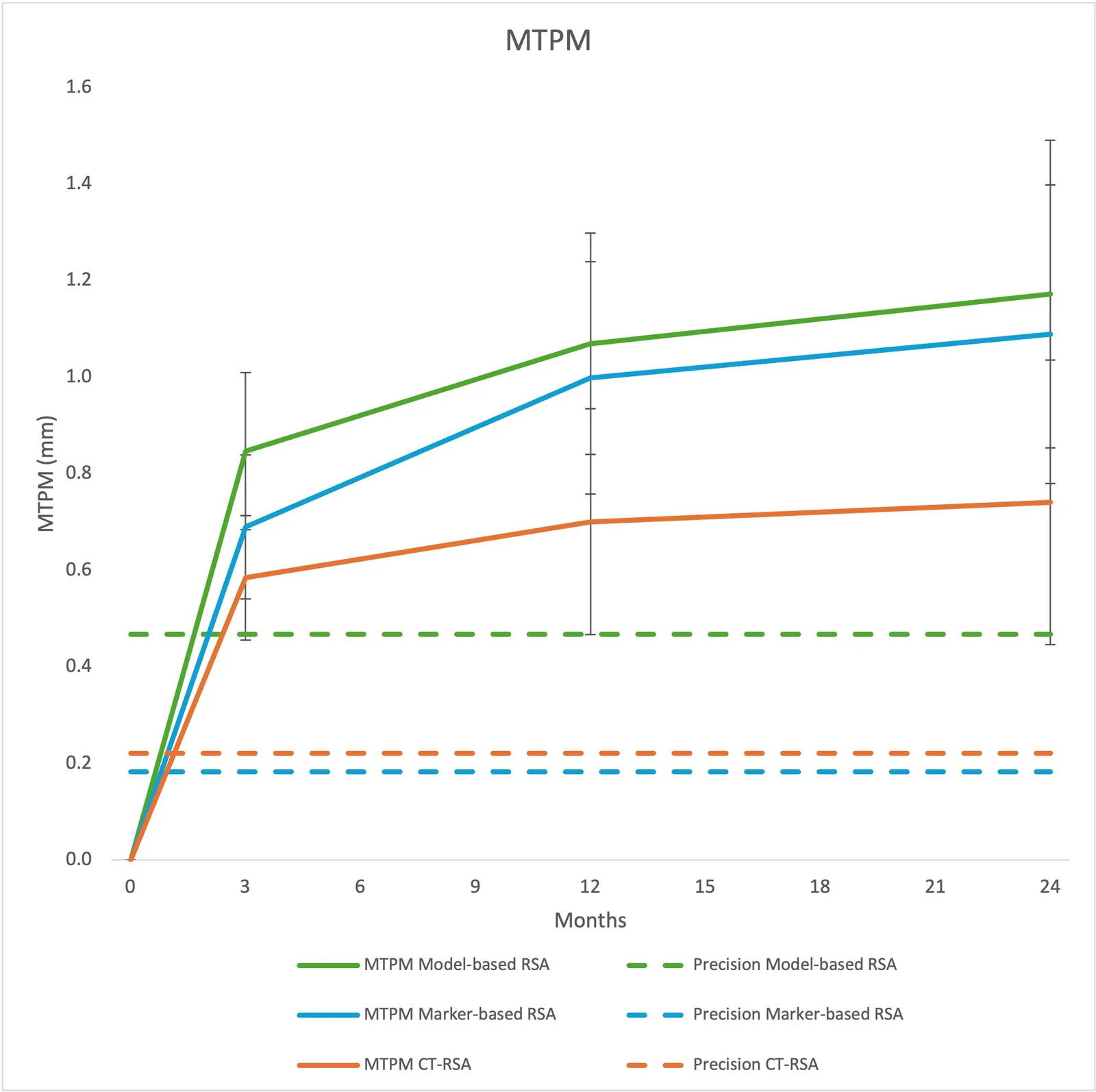
Maximum total point motion (MTPM) for marker-based and model-based radiostereometric analysis (RSA), and CT-based RSA. The figure shows migration up to 24 months with 95% CI (whiskers) and mean precision thresholds (dotted lines, same colour scheme).

**Fig. 6:**
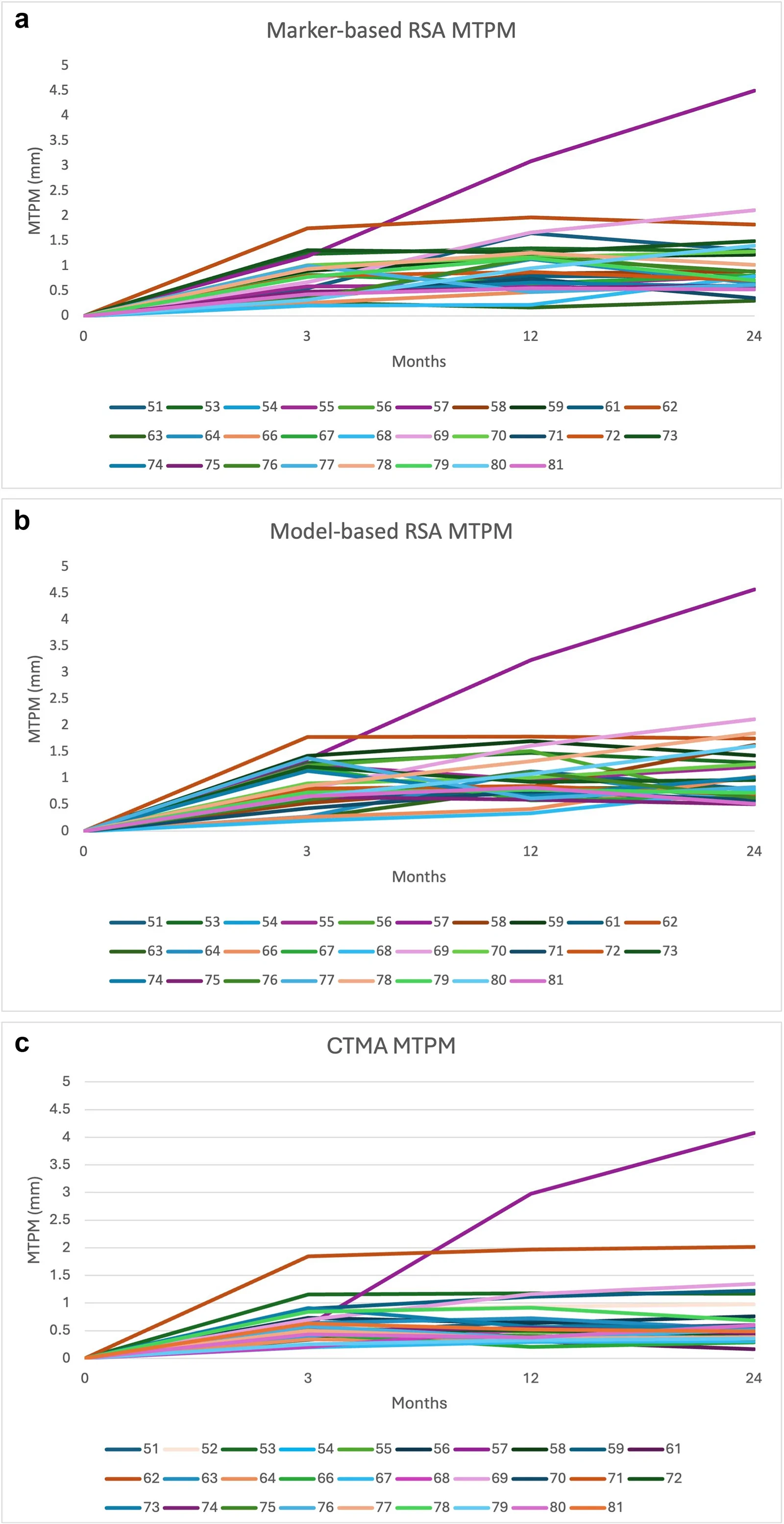
a) Multiple line plot (‘spaghetti plot’) of all analyzed patients (patient IDs 51 to 81) up to 24 months using marker-based radiostereometric analysis (RSA) (n = 28), b) model-based RSA (n = 28), and c) CT-based RSA (n = 29). MTPM, maximum total point motion.

**Table VI.** Migration values for peripheral points in model-based radiostereometric analysis (RSA) and CT-based RSA at 24 months (n = 29 patients).

Variable	Model-based RSA	CT-RSA	p-value*
**Mean tip translation, mm (95% CI)**			
Medial	0.04 (–0.05 to 0.14)†	0.12 (0.04 to 0.19)	0.045
Proximal	–0.21 (–0.33 to –0.08)	–0.12 (–0.21 to –0.04)	0.076
Anterior	0.21 (–0.02 to 0.45)	0.12 (–0.14 to 0.38)	0.230
**Mean medial translation, mm (95% CI)**			
Medial	0.04 (–0.13 to 0.22)	0.10 (0.06 to 0.14)	0.297
Proximal	–0.22 (–0.45 to 0.00)†	–0.08 (–0.20 to 0.05)	0.049
Anterior	0.12 (–0.16 to 0.41)	0.22 (0.08 to 0.37)	0.383
**Mean lateral translation, mm (95% CI)**			
Medial	0.03 (–0.10 to 0.17)	0.10 (0.06 to 0.14)	0.117
Proximal	–0.23 (–0.41 to –0.06)	–0.20 (–0.31 to –0.08)	0.625
Anterior	–0.08 (–0.38 to 0.21)	–0.24 (–0.34 to –0.13)	0.142
**Mean anterior translation, mm (95% CI)**			
Medial	–0.03 (–0.24 to 0.19)	–0.03 (–0.1 to 0.04)	0.960
Proximal	–0.08 (–0.26 to 0.09)	–0.07 (–0.20 to 0.06)	0.879
Anterior	0.05 (–0.13 to 0.23)	–0.01 (–0.07 to 0.04)	0.251
**Mean posterior translation, mm (95% CI)**			
Medial	0.10 (–0.09 to 0.28)‡	0.28 (0.18 to 0.38)	0.012
Proximal	–0.36 (–0.66 to –0.05)	–0.25 (–0.55 to 0.05)	0.291
Anterior	0.01 (–0.21 to 0.20)	0.00 (–0.06 to 0.05)	0.977

* Calculated using a linear mixed model.

† p < 0.050 compared with CT-based RSA.

‡ p < 0.010 compared with CT-based RSA.

Bland–Altman plots for MTPM at 24 months showed overall good agreement between RSA methods, but CT-based RSA showed lower migration values when comparing with both RSA methods (Figure 7). However, the mean difference was stable and there was uniform agreement between the methods.

**Fig. 7:**
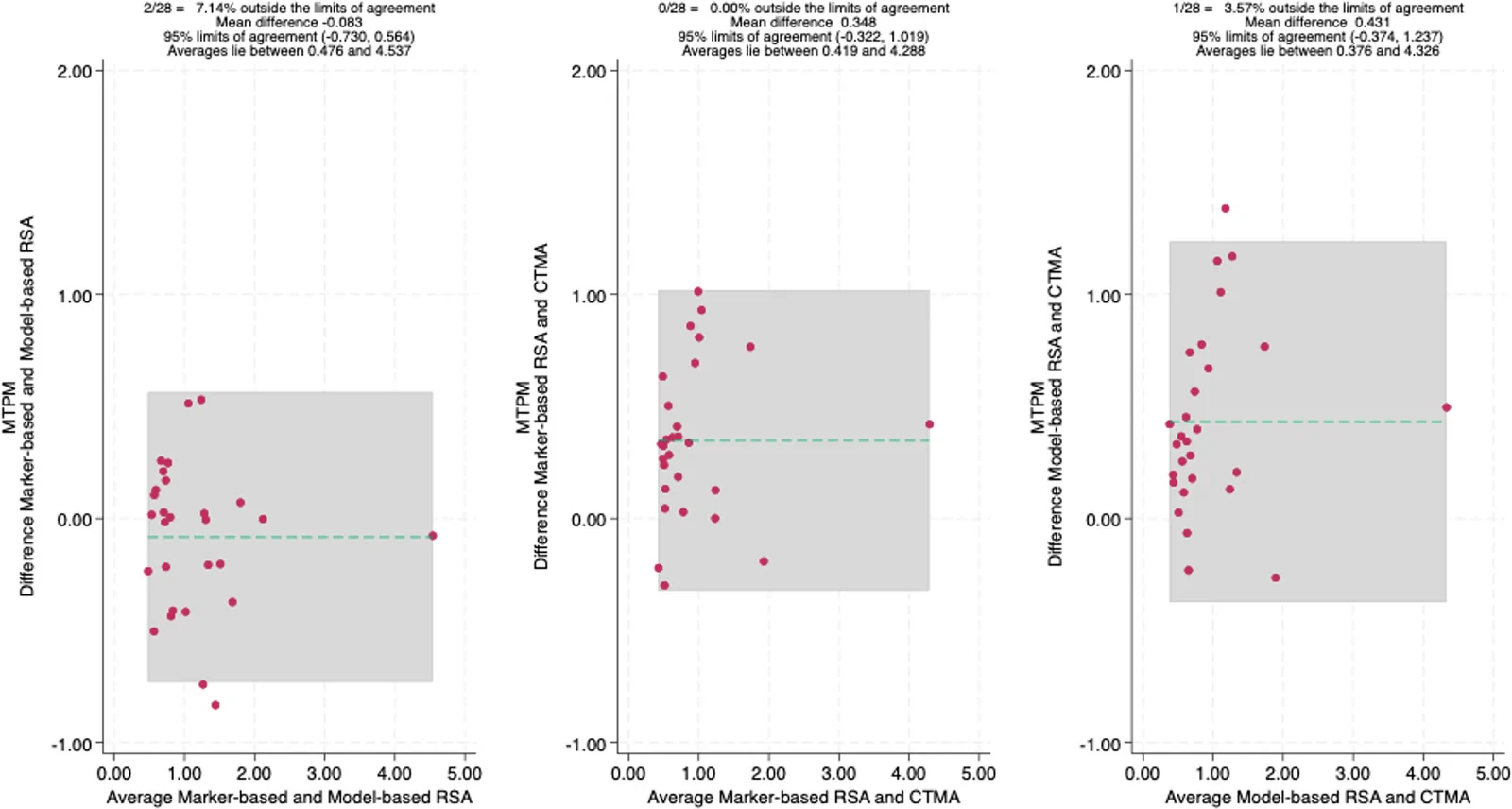
Bland–Altman plot of maximum total point motion (MTPM) at 24 months comparing all three methods. The x-axis describes the average measurements of two methods in relation to the differences between the same measurements (y-axis). The 95% limits of agreement are shown as grey marked area. Mean difference is shown as a dotted green line. CTMA, CT-based micromotion analysis; RSA, radiostereometric analysis.

### Clinical data

ROM, FJS-12, and KOOS scores showed an overall improvement from baseline (preoperatively) to 24 months (Table VII and Figure 8).

**Table VII.** Clinical data at baseline before surgery and at 24 months.

Variable	Mean 24 months (95% CI)	Mean baseline (95% CI)	Mean difference (95% CI)	p-value*
**Range of motion**				
Flexion, °	120.93 (116.78 to 125.07)	108.52 (102.69 to 114.35)	12.41 (5.57 to 19.25)	0.001
Extension lag, °	0.00 (0.00 to 0.00)	2.96 (1.12 to 4.80)	−2.96 (−4.80 to −1.12)	0.003
Flexion contracture, °	0.00 (0.00 to 0.00)	2.67 (0.59 to 4.75)	−2.67 (−4.75 to −0.58)	0.014
HKA axis, °	0.59 (0.11 to 1.07)	3.85 (2.38 to 5.33)	−3.26 (4.73 to −1.78)	< 0.001
ML stability, grade 1 to 3†	1.44 (1.24 to 1.65)	1.37 (1.15 to 1.59)	0.07 (−0.25 to 0.40)	0.646
AP stability, grade 1 to 3†	1.11 (0.98 to 1.24)	1.11 (0.98 to1.24)	0.00 (−0.19 to 0.19)	> 0.999
FJS-12	70.4 (61.5 to 79.4)	12.3 (7.0 to 17.7)	58.1 (47.3 to 68.9)	< 0.001
Pain	86.7 (81.9 to 91.5)	38.8 (33.3 to 44.3)	47.9 (40.4 to 55.4)	< 0.001
Symptoms	83.8 (77.2 to 90.4)	45.0 (37.8 to 52.2)	38.8 (30.0 to 47.6)	< 0.001
ADL	89.0 (84.5 to 93.6)	48.0 (42.4 to 53.6)	41.1 (33.8 to 48.3)	< 0.001
Sport	62.4 (54.0 to 70.8)	15.5 (7.8 to 23.3)	46.9 (35.5 to 58.3)	< 0.001
QoL	79.1 (72.3 to 85.9)	24.8 (20.2 to 29.3)	54.3 (46.6 to 62.0)	< 0.001
KOOS-Total	82.7 (78.4 to 87.1)	42.5 (38.7 to 46.2)	40.3 (34.4 to 46.1)	< 0.001

Statistical t-test was utilized for clinical data p-values.

* Calculated using paired *t*-tests.

† Grade 1: < 5 mm laxity, Grade 2: 5 mm to 10 mm laxity, Grade 3: > 10 mm laxity.

ADL, activities of daily living; AP, anteroposterior; FJS-12, Forgotten Joint Score-12; HKA, hip-knee-ankle axis; KOOS, Knee Injury and Osteoarthritis Score; KSS, Knee Society Score; ML, mediolateral; QoL, quality of life.

**Fig. 8:**
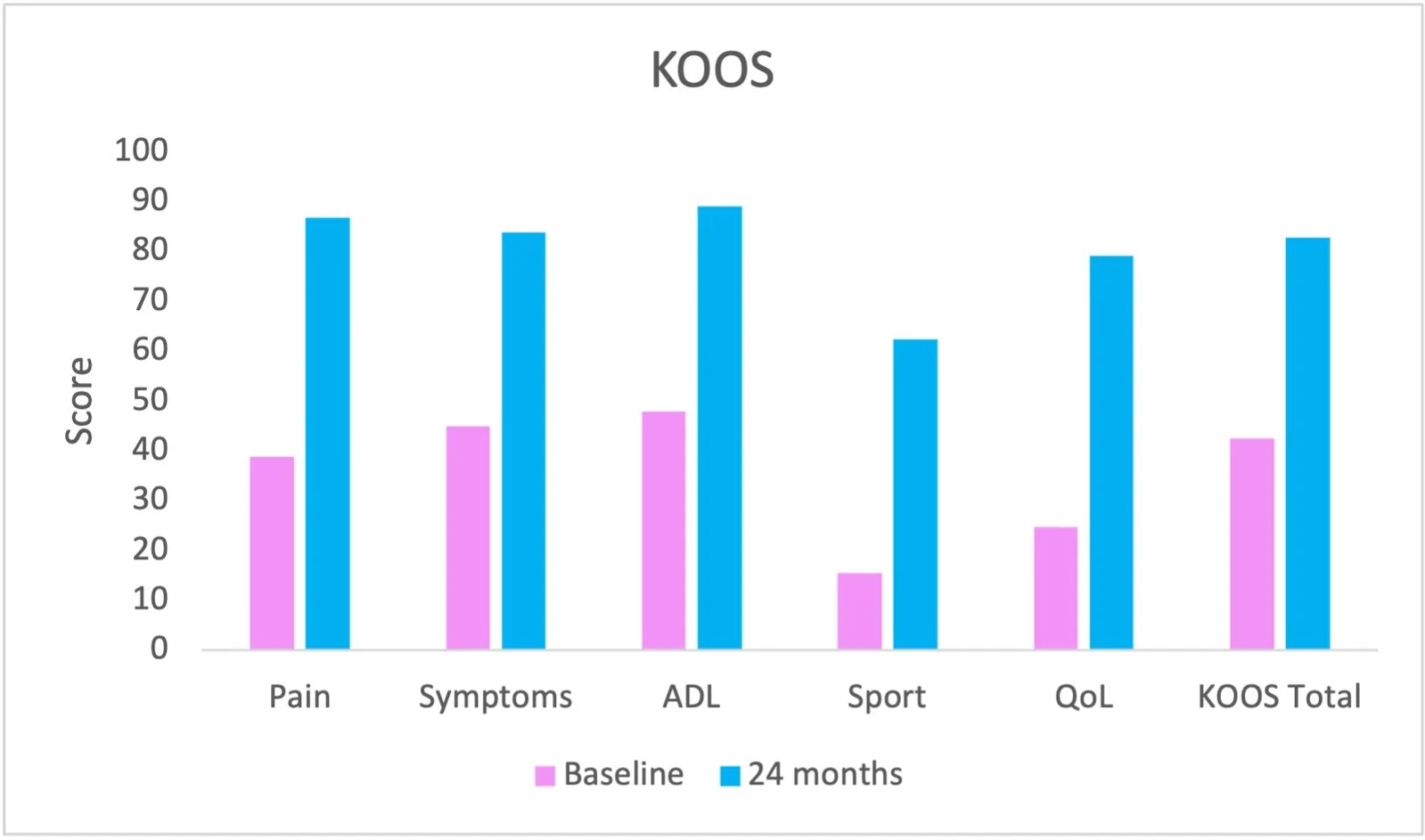
Knee Injury and Osteoarthritis Outcome Score (KOOS) sub-scale scores measured preoperatively at baseline and at 24 months. ADL, activities of daily living; QoL, quality of life.

## Discussion

In this study we found that the GMK Sphere MP tibial implant was stable up to two years. All methods showed stable migration patterns, although CT-based RSA showed lower values. The lower migration values of CT-based RSA for MTPM at 12 months were also shown at 24 months (Table V and Figure 5).^10^ Current accuracy studies show both accuracy and precision of CT-based RSA alternatives that are at the same level or better than RSA.^8,9^ The questions that arise are therefore which values are correct, whether CT-based RSA values are misleadingly low, and how this affects the known limit of safe migration based solely on RSA.^3^

It can be argued that lower CT-based RSA migration values are real, as RSA utilizes biplanar radiographs leading to lower precision and accuracy than CT. Work by de Laat et al^32^ has raised the possibility that lower instrument noise could explain why CT-based RSA shows lower MTPM. One hypothesis is that since CT-based RSA is a true 3D method, there is less instrument noise than RSA which uses two dimensions to construct a third. Segmental migration confidence intervals had a smaller spread for CT-RSA methods than RSA methods, possibly signifying a lower level of instrument uncertainty for CT-RSA (Table V). Additionally, in the same study by de Laat et al,^32^ a cemented TKA showed a lower MTPM with CT-based RSA than RSA, but the difference was not statistically significant. However, since our study tested an uncemented TKA, it is likely that the difference observed in this study is larger due to the higher absolute values of MTPM shown by uncemented implants, leading to larger measurement uncertainties. Nonetheless, despite the differences in MTPM, safe levels of migration were shown across all methods.^3^

The question of why MBRSA does not show external rotation compared with CT-based RSA and marker-based RSA can be answered by MBRSA’s low sensitivity for internal rotation and low precision (Table V).^10,22^ Difficulties with setting the CAD model’s rotation are avoided using marker-based RSA and CT-based RSA. Furthermore, the medial translation shown by CT-based RSA, and not RSA, is not large enough to be considered meaningful, despite its statistically significant p-value (Table V). The medial translations also showed partially overlapping CIs, reinforcing the clinical insignificancy of this discrepancy.

RSA has high precision and accuracy, but can only be used as a research tool. However, CT is readily available, and CT-based RSA software is available upon purchase of one of two options (Ortoma treatment Solutions (Ortoma AB, Sweden) and CTMA (Sectra AB, Sweden)). This shows the potential of CT-based RSA implementation in migration analysis of implants. As such, accuracy studies should be undertaken to determine which method is correct.

Effective radiation doses were 0.010 mSv for RSA and 0.071 mSv for CT. CT examinations expose patients to 5.3 times higher radiation than RSA (Table II), but this only equals 5.8 days of background radiation in Norway.^33^ These lower doses do not reduce CT-based RSA precision.^34^ In clinical RSA studies, radiograph retakes due to poor-quality exposures of tantalum beads are common. Retakes of CT examinations are not necessary, as CT-RSA does not require tantalum beads. Although loosening of the femur component in TKA is rare, RSA studies are difficult to perform due to the shape of the femoral implant and obscured tantalum beads, while CT-based RSA is possible for femoral implants.^35^

In migration analysis, RSA provides the user with quality markers. CTMA software uses surface anatomy for calculations of implant migration and does not have numbered values that are comparable with RSA. CTMA uses a heat map for segmentation and posing quality. It is challenging to accurately determine if CTMA has been performed correctly. However, comparable precision between CT-based RSA and RSA methods,^10^ and high intraclass corelation coefficient (ICC) between evaluators, indicate a robust method.^7^ Furthermore, Sectra requires users to be approved before use, and the first author (LE) has extensive experience using CT-based RSA.^7,10^

Migration of the uncemented GMK Sphere tibial implant using mechanical alignment with conventional instruments at 12 months is well below the acceptable cut-off value of 1.10 mm at six to 12 months.^3^ Additionally, the implant also shows safe migration from 12 to 24 months. Furthermore, known limits for safe migration based on RSA studies are difficult to use with values obtained by CT-based RSA, as CT-based RSA seems to measure lower values. However, the uncemented MP tibial implant showed safe migration using all methods.

According to papers by Laende et al^36^ and Niesen et al,^37^ the use of mechanical alignment compared with kinematic alignment produces no difference in migration. Therefore, it is likely that the safety of this uncemented MP tibial implant is also valid with the use of kinematic alignment. However, this was not specifically tested in this study. Alternatively, the limits of safe migration were built on studies preceding personalized alignment strategies, and therefore these thresholds may only apply to MA. HKA, MPTA, and Berger’s rotation showed sound values for implant positioning and survival. Furthermore, clinical data changed from baseline to 24 months, in keeping with previous studies for KOOS,^38^ FJS-12,^38^ and ROM,^39^ which further strengthens the results.

This study had several strong points. Gold-standard RSA migration methods, along with supportive clinical data, were used in evaluation of the new implant’s safety. Surgical and radiological quality control measures were analyzed and included. RSA and CT-based RSA were directly compared for one TKA up to 24 months, including precision. However, this study does not have the ability to prove whether CT-based RSA is similar in accuracy to the gold-standard RSA. Thus, these findings cannot fully validate the clinical use of CT-based RSA as a substitution for conventional RSA; further accuracy studies are necessary. To that end, phantom studies are currently in progress for knee, hip, shoulder, and ankle prostheses, and should help to determine the validation of CT-based RSA compared with RSA. Questions that this study has raised, but not answered, are why CT-based RSA shows lower migration values than RSA methods, and whether similar safe levels of migration would be observed with kinematic alignment or non-conventional instruments.

In conclusion, migration of the uncemented GMK Sphere medial pivot tibial implant shows safe migration at six and 12 months, and stable patterns of migration from 12 to 24 months, using all methods. CT-based RSA shows lower migration than RSA methods, but the difference is not of clinical importance in this study. The uncemented GMK Sphere should therefore be defined as a safe implant at 24 months.

**Take home message**

- CT-based radiostereometric analysis (CT-RSA) is quicker and as precise as RSA methods and could therefore be used in evaluation of migration of tibial implants.

- CT-RSA requires less equipment and does not require surgically implanted tantalum beads and therefore has the potential to revolutionize implant migration analysis.

- The GMK Sphere total knee arthoplasty shows safe migration values using CT-RSA and conventional radiostereometric analysis

## Data Availability

The data that support the findings for this study are available to other researchers from the corresponding author upon reasonable request.
